# Posterior tibial slope is associated with ACL status rather than demographic factors: A cohort of 1406 patients

**DOI:** 10.1002/jeo2.70890

**Published:** 2026-08-18

**Authors:** Julien Druel, Romir Patel, Christophe Jacquet, Lyes Chaal, Antoine Piercecchi, Matthieu Ollivier

**Affiliations:** ^1^ Department of Orthopedic Surgery, Assistance Publique des Hôpitaux de Marseille, Institute for Locomotion, Hôpital Sainte Marguerite, St Marguerite Hospital Aix‐Marseille University Marseille France; ^2^ Department of Biomechanics APHM, CNRS, ISM, St Marguerite Hospital, Institute for Locomotion Aix‐Marseille University Marseille France

**Keywords:** ACL graft failure, anatomical risk factor, anterior cruciate ligament injury, knee biomechanics, posterior tibial slope, tibial slope

## Abstract

**Purpose:**

To determine whether posterior tibial slope (PTS), measured on lateral radiographs using the proximal anatomical tibial axis defined by two circles, is independently associated with anterior cruciate ligament (ACL) injury status or influenced by age, sex and body mass index (BMI).

**Methods:**

A retrospective single‐centre cohort study included all 1406 consecutive eligible patients operated between January 2010 and September 2024: 857 ACL‐intact controls undergoing medial patellofemoral ligament reconstruction, 498 patients with primary ACL rupture and 51 patients with ACL re‐rupture. PTS was measured on standardized lateral radiographs using the proximal anatomical tibial axis defined by two circles. Multivariable linear regression evaluated the independent associations of ACL status, age, sex and BMI with PTS. Receiver operating characteristic (ROC) analyses assessed discrimination of ACL injury.

**Results:**

Mean PTS was 7.3 ± 3.1° in controls, 9.9 ± 3.1° in patients with primary ACL rupture and 10.9 ± 3.5° in patients with ACL re‐rupture (*p* < 0.001). Intraobserver and interobserver reliability were good to excellent (intraclass correlation coefficient [ICC] = 0.88) and moderate to good (ICC = 0.81). Multivariable analysis demonstrated that ACL injury status was the dominant independent determinant of PTS. Compared with controls, primary ACL rupture and ACL re‐rupture were associated with 2.52° and 3.63° greater PTS, respectively (both *p* < 0.001). Age showed a minimal inverse association (−0.019° per year; 95% confidence interval [CI], −0.036° to −0.003°; *p* = 0.024), whereas sex and BMI were not independently associated with PTS. ROC analysis identified a Youden‐derived value of 10°, with an area under the curve of 0.731 (95% CI, 0.705–0.758) for primary ACL rupture and 0.791 (95% CI, 0.741–0.841) for ACL re‐rupture.

**Conclusion:**

PTS was independently associated with ACL injury and ACL re‐rupture, whereas age, sex and BMI had no clinically meaningful independent influence. PTS should be interpreted as one component of a multifactorial anatomical risk profile rather than as a standalone screening test.

**Level of Evidence:**

Level III, retrospective comparative cohort study.

## Graphical abstract

Conventional posterior tibial slope values and principal adjusted findings.

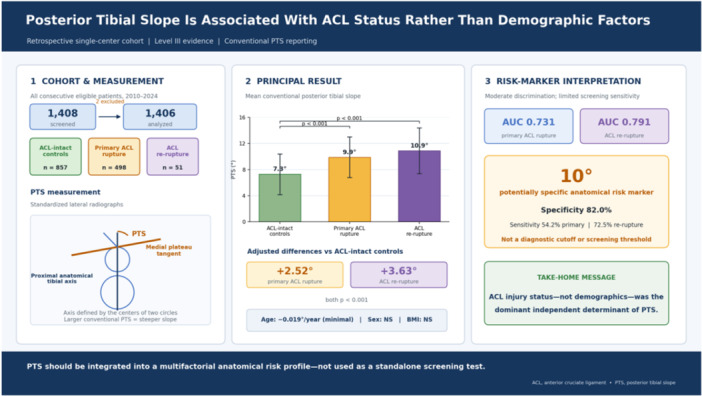

**Abbreviations**
*β*
: standardized regression coefficient
ACL: anterior cruciate ligament
ACLR: anterior cruciate ligament reconstruction
AUC: area under the curve
BMI: body mass index
CI: confidence interval
ICC: intraclass correlation coefficient
IRB: Institutional Review Board
MDC: minimal detectable change
MPFL: medial patellofemoral ligament
MRI: magnetic resonance imaging
OLS: ordinary least squares
PTS: posterior tibial slope
ROC: receiver operating characteristic
SD: standard deviation
SEM: standard error of measurement
TT–TG: tibial tubercle–trochlear groove

## INTRODUCTION

Posterior tibial slope (PTS) is a well‐established anatomical risk factor for anterior cruciate ligament (ACL) injury and graft failure [6]. A steeper PTS increases anterior tibial translation under axial loading and therefore increases ACL loading during weight‐bearing and pivoting activities. Experimental, biomechanical and clinical evidence has consistently associated increased PTS with both primary ACL rupture and subsequent graft failure after ACL reconstruction [11, 13, 17, 21]. Consequently, PTS assessment has become an important component of the anatomical evaluation of patients undergoing ACL reconstruction [9, 18].

PTS can be measured on lateral radiographs or magnetic resonance imaging using mechanical or anatomical tibial reference axes [2, 10]. Measured values and proposed risk thresholds vary according to the selected imaging modality, reference axis and measurement technique [4]. In the present study, PTS was measured on lateral radiographs using the proximal anatomical tibial axis defined by the centres of two circles fitted within the proximal tibia [7, 20]. All values were converted and are reported according to the conventional PTS definition, in which larger values indicate a steeper posterior slope.

Although the association between increased PTS and ACL injury is well established, whether age, sex or body mass index (BMI) independently influences PTS remains uncertain. Demographic effects could require different reference values during preoperative risk assessment. Previous studies have included selected populations and have reported inconsistent associations between demographic characteristics and PTS [2, 5, 8, 14].

No previous large cohort was identified that simultaneously evaluated the independent associations of ACL injury status, age, sex and BMI with PTS in ACL‐intact controls, patients with primary ACL rupture and patients with ACL re‐rupture.

The aim of this study was to determine the independent determinants of PTS measured on lateral radiographs using the proximal anatomical tibial axis defined by two circles in a large cohort of ACL‐intact controls, patients with primary ACL rupture and patients with ACL re‐rupture. It was hypothesized that ACL injury status would be the principal independent determinant of PTS, whereas age, sex and BMI would have no clinically meaningful independent association after multivariable adjustment. It was also hypothesized that PTS would discriminate ACL‐intact from ACL‐injured knees.

## METHODS

### Study design and patient selection

A retrospective cohort study was conducted at a single tertiary referral centre specializing in knee surgery and ligament reconstruction. All consecutive patients who underwent surgery between January 2010 and September 2024 were screened for eligibility.

Three groups were defined a priori:

1. Patients with an intact ACL undergoing medial patellofemoral ligament (MPFL) reconstruction for recurrent patellar instability (control group).
2. Patients undergoing primary ACL reconstruction following complete ACL rupture.
3. Patients undergoing revision ACL reconstruction following graft failure.

The MPFL reconstruction group was selected as the surgical control population because the procedure does not involve the ACL or alter tibial morphology and systematic preoperative imaging was available. ACL integrity was confirmed on preoperative MRI by two independent musculoskeletal radiologists. Patients with a partial or complete ACL tear, mucoid degeneration or a bone‐bruise pattern suggestive of ACL injury were excluded. Arthroscopic confirmation was not routinely available because diagnostic arthroscopy is not systematically performed during isolated MPFL reconstruction. Patellofemoral abnormalities such as trochlear dysplasia or increased tibial tubercle–trochlear groove distance do not alter the proximal tibial reference axis used for PTS measurement [2, 7, 20].

Additional exclusion criteria included previous proximal tibial osteotomy, severe post‐traumatic deformity, congenital skeletal abnormalities affecting the proximal tibia, advanced bone loss preventing reliable identification of the tibial plateau and inadequate radiographs precluding accurate PTS measurement.

The study was approved by the Institutional Review Board (IRB approval No. CSE_PADS25_044_DGR) and conducted in accordance with the Declaration of Helsinki. The requirement for informed consent was waived because of the retrospective design and anonymized data collection.

### Radiographic assessment

PTS was measured exclusively on standardized true lateral knee radiographs. Before measurement, all radiographs were independently reviewed by two fellowship‐trained orthopaedic surgeons to assess their suitability for PTS analysis.

Only radiographs fulfilling predefined quality criteria were included. An adequate lateral radiograph was defined as complete visualization of the proximal tibia and tibial plateau with satisfactory superimposition of both the posterior femoral condyles and the distal femoral condyles, allowing reliable identification of the tibial plateau and proximal anatomical tibial axis. Radiographs demonstrating excessive rotational malalignment, inadequate femoral condylar overlap, incomplete visualization of the proximal tibia, poor image quality or projection errors were considered inadequate and excluded from analysis.

These criteria were applied because rotational malpositioning and inadequate condylar superimposition on lateral radiographs can influence PTS measurements and reduce reliability [4, 7].

### PTS measurement

The proximal anatomical tibial axis was defined by connecting the centres of two circles fitted within the proximal tibia, as described for radiographic PTS assessment [2, 7, 20]. A tangent was drawn along the medial tibial plateau. Conventional PTS was calculated as the angle between this tangent and a line perpendicular to the proximal anatomical tibial axis (90° minus the originally recorded inverse angle); therefore, larger values indicate a steeper posterior slope.

The measured PTS ranged from −1.0° to 19.8°.

All measurements were performed digitally using standardized PACS software by two fellowship‐trained orthopaedic surgeons who were blinded to clinical group allocation.

To assess reproducibility, a random sample of 60 radiographs was re‐evaluated.

Intraobserver and interobserver reliability were assessed using intraclass correlation coefficients (ICCs; two‐way mixed‐effects model, absolute agreement). Following the recommendations of Koo and Li [12], reliability categories were interpreted according to the lower bound of the 95% confidence interval (CI) rather than the point estimate alone. Accuracy against an external reference standard was not assessed. Because no absolute measurement‐error statistic was available, the standard error of measurement, minimal detectable change and limits of agreement were not reported.

### Covariates

The following variables were collected at the time of surgery: age, sex, BMI and ACL injury status (control, primary ACL rupture or revision ACL reconstruction).

Age was analysed as both a continuous variable and a categorical variable (<18 years vs. ≥18 years), the latter corresponding to the conventional distinction between adolescent and adult patients in orthopaedic research.

BMI data were unavailable for 384 patients treated during the early study period. BMI‐adjusted complete‐case analyses therefore included 1022 patients (72.7% of the cohort). No additional observations were omitted from the full‐cohort analysis.

Activity level (e.g., Tegner Activity Scale) was not routinely recorded throughout the study period and was therefore unavailable for analysis.

### Statistical analysis

Continuous variables were reported as mean ± standard deviation (SD), and categorical variables were reported as frequencies and percentages.

Between‐group comparisons were performed using one‐way analysis of variance (ANOVA) or the Kruskal–Wallis test as appropriate. Pairwise comparisons were performed using Welch's *t* test or Mann–Whitney *U* test with Bonferroni correction.

The independent associations between PTS and ACL injury status, age, sex and BMI were evaluated using multivariable ordinary least‐squares linear regression.

Two predefined regression models were constructed:

- Model 1: Age, sex and ACL status (entire cohort).
- Model 2: Age, sex, ACL status and BMI (complete‐case cohort).

Regression coefficients, standardized *β* coefficients, 95% CIs, and *p* values are reported.

Receiver operating characteristic (ROC) analyses were used to evaluate the ability of PTS to discriminate ACL‐injured knees from ACL‐intact controls. A Youden‐derived value was calculated for descriptive purposes; it was not interpreted as a diagnostic or screening cutoff.

Because this retrospective cohort included all consecutive eligible patients during the study period, no prospective a priori sample‐size calculation was performed. Precision was evaluated using effect sizes and 95% CIs.

Statistical analyses were performed using Python version 3.10 (Python Software Foundation) with SciPy (v1.11.4), statsmodels (v0.14.1) and scikit‐learn (v1.3.2). Statistical significance was set at *p* < 0.05.

## RESULTS

### Study population

**Figure 1:**
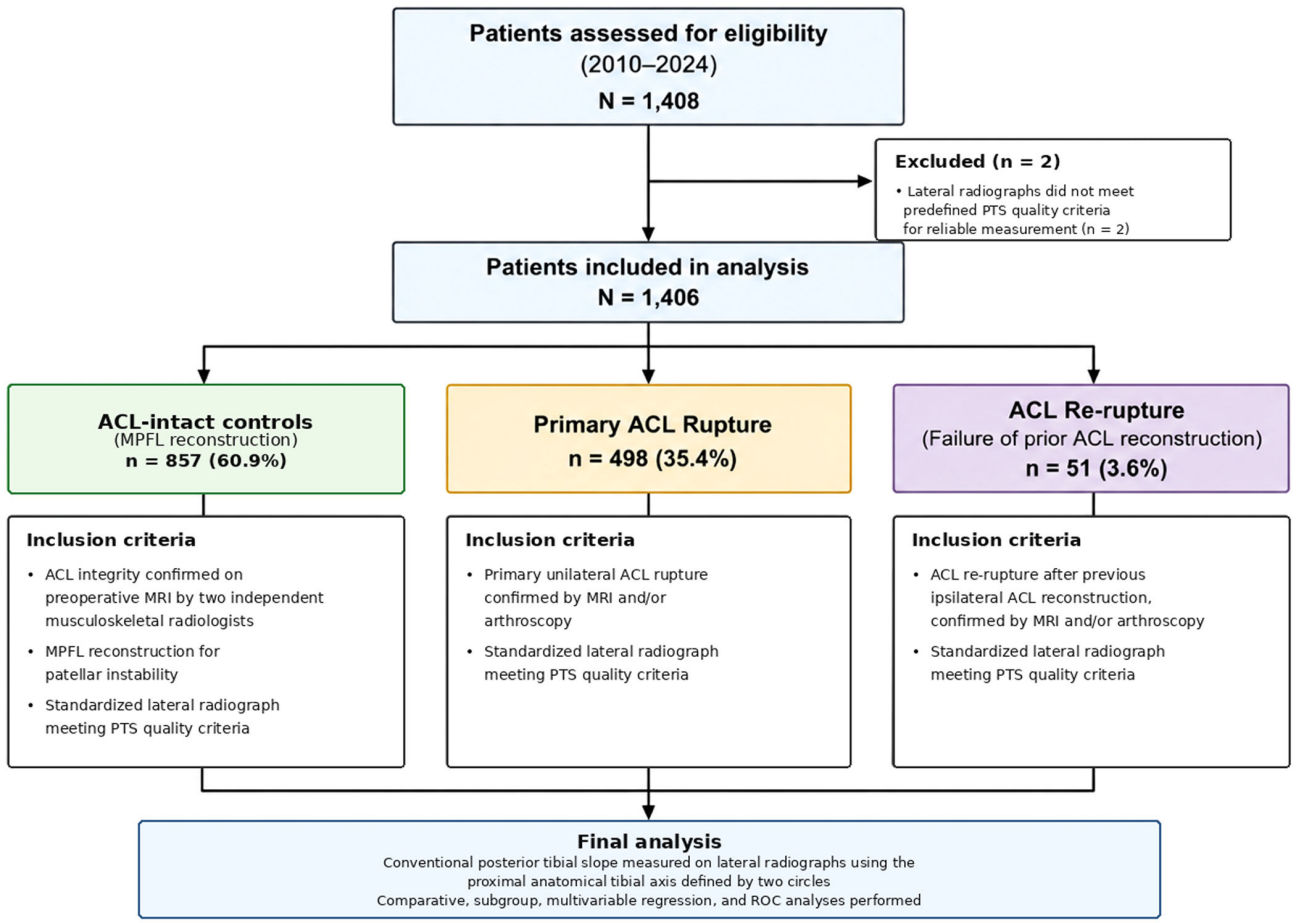
Flowchart of patient selection and cohort allocation. Flowchart illustrating patient selection and inclusion in the study. Between January 2010 and September 2024, 1408 consecutive patients were screened for eligibility. Two patients were excluded because the available lateral radiographs did not meet the predefined quality criteria for reliable posterior tibial slope (PTS) measurement. The final study population comprised 1406 patients: 857 ACL‐intact controls undergoing medial patellofemoral ligament reconstruction, 498 patients with primary ACL rupture and 51 patients with ACL re‐rupture. Conventional PTS was measured on lateral radiographs using the proximal anatomical tibial axis defined by two circles. ACL, anterior cruciate ligament.

A total of 1408 consecutive patients were screened for eligibility. Two patients were excluded because their lateral radiographs did not meet the predefined quality criteria for reliable PTS measurement, leaving 1406 patients for the final analysis (Figure 1). The final cohort comprised 857 ACL‐intact controls undergoing MPFL reconstruction, 498 patients with primary ACL rupture and 51 patients with ACL re‐rupture.

The mean age of the cohort was 27.2 ± 10.2 years (range, 7–50 years), and 45.2% were male. Patients with primary ACL rupture were younger than controls (23.4 ± 9.6 vs. 29.2 ± 10.1 years), whereas patients undergoing revision ACL reconstruction were older (31.0 ± 5.1 years). Because of these baseline differences, age was included as a covariate in all multivariable analyses. Baseline demographic and clinical characteristics are summarized in Table 1.

**Table 1:** Baseline demographic and clinical characteristics of the study population.

Variable	Full cohort (*N* = 1406)	ACL‐intact controls (MPFL) (*n* = 857)	Primary ACL rupture (*n* = 498)	ACL re‐rupture (*n* = 51)	*p* value
Age (years)	27.2 ± 10.2	29.2 ± 10.1	23.4 ± 9.6	31.0 ± 5.1	<0.001
Age <18 years, *n* (%)	352 (25%)	162 (19%)	189 (38%)	0 (0%)	<0.001
Sex: Female, *n* (%)	772 (55%)	501 (58%)	249 (50%)	22 (43%)	0.007
BMI (kg/m^2^)	24.0 ± 4.5	24.4 ± 4.6	23.1 ± 4.3	24.8 ± 4.6	0.001
BMI available, *n*	1022	611	385	26	
Conventional PTS (°): Mean ± SD	8.4 ± 3.4	7.3 ± 3.1	9.9 ± 3.1	10.9 ± 3.5	<0.001
Median (IQR)	8.3 (6.0–10.5)	7.4 (5.2–9.4)	10.0 (8.0–12.0)	11.9 (9.6–13.4)	
Range	−1.0–19.8	−1.0–19.8	0.0–18.4	1.7–16.2	

*Note*: Baseline demographic and clinical characteristics of ACL‐intact controls, primary ACL rupture patients and ACL re‐rupture patients. Continuous variables are presented as mean ± SD and categorical variables as number (percentage). Group comparisons were performed using one‐way ANOVA or Kruskal–Wallis tests for continuous variables and *χ* ^2^ tests for categorical variables, as appropriate.

ACL re‐rupture group: Adults only (0 patients aged < 18 years). BMI was unavailable for 384 of 1406 patients (27.3%).

Values are mean ± SD, median (IQR), range or *n* (%). Larger conventional PTS values indicate a steeper posterior slope. *p* values: One‐way ANOVA, Kruskal–Wallis test or *χ* ^2^ test, as appropriate.

Abbreviations: ACL, anterior cruciate ligament; ANOVA, analysis of variance; BMI, body mass index; IQR, interquartile range; MPFL, medial patellofemoral ligament; PTS, posterior tibial slope; SD, standard deviation.

### PTS according to ACL status

Mean PTS for the full cohort was 8.4 ± 3.4° (range, −1.0° to 19.8°).

**Figure 2:**
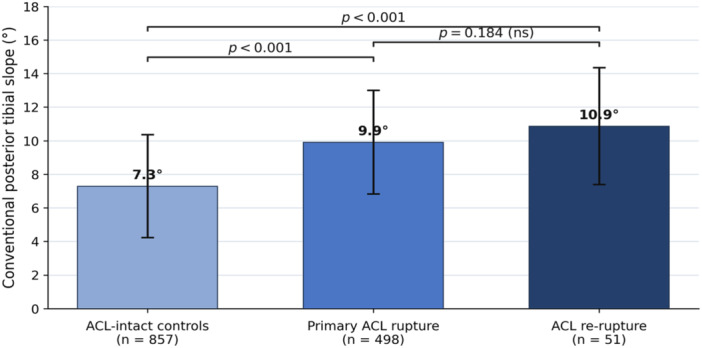
Conventional PTS according to ACL injury status. Mean conventional PTS in ACL‐intact controls, patients with primary ACL rupture and patients with ACL re‐rupture. Error bars represent standard deviations. Horizontal brackets indicate Bonferroni‐adjusted pairwise comparisons between groups. The PTS was significantly greater in the primary ACL rupture and ACL re‐rupture groups than in ACL‐intact controls (both *p* < 0.001), whereas the difference between the primary ACL rupture and ACL re‐rupture groups was not significant (*p* = 0.184; Welch *t* test). Larger values indicate a steeper posterior tibial slope. ACL, anterior cruciate ligament; PTS, posterior tibial slope.

PTS differed significantly among the three study groups (one‐way ANOVA, *p* < 0.001; Kruskal–Wallis, *p* < 0.001). Mean PTS was 7.3 ± 3.1° in ACL‐intact controls, 9.9 ± 3.1° in patients with primary ACL rupture and 10.9 ± 3.5° in patients with ACL re‐rupture (Figure 2).

Compared with controls, conventional PTS was 2.6° greater in patients with primary ACL rupture (95% CI, 2.3°–3.0°; *p* < 0.001; Cohen's *d* = 0.85) and 3.6° greater in patients with ACL re‐rupture (95% CI, 2.6°–4.5°; *p* < 0.001; Cohen's *d* = 1.09).

The 1.0° difference between primary ACL rupture and ACL re‐rupture was not significant after Welch's *t* test with Bonferroni correction (*p* = 0.184), although the Mann–Whitney *U* test was significant (*p* = 0.008). This finding should be interpreted cautiously given the limited size of the re‐rupture group (Table 2).

**Table 2:** Distribution and pairwise comparisons of conventional PTS between study groups.

A. Distribution of conventional PTS (D'Agostino–Pearson *K* ^2^ test)								
Group	*N*	Mean PTS (°)	SD (°)	Skewness	Kurt.	*K* ^2^	*p* value	Interpretation
ACL‐intact controls (MPFL)	857	7.30	3.07	0.162	0.283	6.623	0.069	Normal (*p* ≥ 0.05)
Primary ACL rupture	498	9.92	3.09	−0.199	−0.074	3.417	0.191	Normal (*p* ≥ 0.05)
ACL re‐rupture	51	10.87	3.48	−0.870	−0.135	6.472	0.072	Normal (*p* ≥ 0.05)
Full cohort	1406	8.36	3.37	0.040	−0.214	3.054	0.217	Normal (*p* ≥ 0.05)
Conventional PTS is reported in degrees; larger values indicate a steeper posterior slope. D'Agostino–Pearson normality test; Welch *t* test and Mann–Whitney *U* test; Bonferroni correction for three comparisons.								

B. Overall between‐group tests for conventional PTS					
**Test**	**Statistic**	**df**	***p* value**	**Effect size**	**Significance**
One‐way ANOVA	*F* = 130.991	2, 1403	<0.001	*η* ^2^ = 0.1573	***
Kruskal–Wallis *H*	*H* = 227.309	2	<0.001	—	***

**C. Post hoc pairwise comparisons of conventional PTS (Bonferroni corrected for three comparisons)**															
**Comparison (group 1 − group 2)**	**n_1_**	**Mean_1_ PTS**	**SD_1_**	**n_2_**	**Mean_2_ PTS**	**SD_2_**	**Δ PTS (°)**	**95% CI (°)**	***t***	***p* (raw)**	***p* (Bonf.)**	**Cohen's *d***	***U***	***p* (MW)**	**Sig.**
Primary ACL rupture − ACL‐intact controls	498	9.92	3.09	857	7.30	3.07	2.625	[2.29, 2.96]	15.114	<0.001	<0.001	0.852	1,14,964	<0.001	***
ACL re‐rupture − ACL‐intact controls	51	10.87	3.48	857	7.30	3.07	3.573	[2.60, 4.55]	7.171	<0.001	<0.001	1.089	9183	<0.001	***
ACL re‐rupture − primary ACL rupture	51	10.87	3.48	498	9.92	3.09	0.948	[−0.04, 1.94]	1.872	0.061	0.184	0.288	9836	0.008	n.s.

Δ PTS = Mean_1 _− Mean_2_. Positive values indicate a greater, steeper conventional PTS in Group 1. Bonferroni significance threshold: *p* < 0.017. *U* is the smaller Mann–Whitney statistic.

*Note*: Conventional PTS was compared among ACL‐intact controls undergoing medial patellofemoral ligament reconstruction, patients with primary ACL rupture and patients with ACL re‐rupture. Larger values indicate a steeper posterior slope. Distributional characteristics, overall one‐way analysis of variance and Kruskal–Wallis tests, and post hoc pairwise comparisons are reported. Pairwise mean differences were calculated as Group 1 minus Group 2 and are presented with 95% CIs, Welch *t* test statistics, Bonferroni‐adjusted *p* values, Cohen's *d* effect sizes and Mann–Whitney *U* test results.

Abbreviations: ACL, anterior cruciate ligament; ANOVA, analysis of variance; CI, confidence interval; MPFL, medial patellofemoral ligament; n.s., not significant; PTS, posterior tibial slope; SD, standard deviation.

Intraobserver reliability was good to excellent (ICC, 0.88; 95% CI, 0.79–0.93), and interobserver reliability was moderate to good (ICC, 0.81; 95% CI, 0.69–0.88). Accuracy against an external reference standard and absolute measurement error were not assessed.

### Multivariable analysis

Multivariable linear regression demonstrated that ACL injury status was the strongest independent determinant of PTS (Table 3).

**Table 3:** Multivariable linear regression analysis of factors associated with conventional PTS.

Variable	*B* (°)	Standardized *β*	SE (°)	95% CI (°)	*t*	*p* value	Sig.
Model 1—Outcome: conventional PTS (°) | Age + Sex + ACL Group (no BMI) | *N* = 1406 | *R* ^2^ = 0.1603 | Adj *R* ^2^ = 0.1579							
Intercept	7.778	—	0.285	[7.219, 8.336]	27.291	<0.001	[***]
Age (years)	−0.019	−0.058	0.008	[−0.036, −0.003]	−2.262	0.024	[*]
Female sex (vs. male)	0.134	0.020	0.166	[−0.192, 0.460]	0.804	0.422	n.s.
ACL rupture (vs. MPFL)	2.520	0.358	0.181	[2.165, 2.876]	13.902	<0.001	[***]
ACL re‐rupture (vs. MPFL)	3.629	0.202	0.446	[2.755, 4.504]	8.135	<0.001	[***]
Outcome: conventional PTS (°); larger values indicate a steeper posterior slope. Reference: MPFL/ACL‐intact group and male sex. OLS.							
Model 2—Outcome: conventional PTS (°) | Age + Sex + BMI + ACL Group | *N* = 1022 | *R* ^2^ = 0.1547 | Adj *R* ^2^ = 0.1505							
Intercept	7.649	–	0.591	[6.490, 8.807]	12.943	<0.001	[***]
Age (years)	−0.032	−0.096	0.010	[−0.052, −0.012]	−3.088	0.002	[**]
Female sex (vs. male)	−0.003	0.000	0.202	[−0.399, 0.392]	−0.017	0.986	ns
BMI (kg/m^2^)	0.015	0.020	0.023	[−0.030, 0.060]	0.656	0.512	ns
ACL rupture (vs. MPFL)	2.486	0.348	0.219	[2.058, 2.915]	11.378	<0.001	[***]
ACL re‐rupture (vs. MPFL)	3.312	0.151	0.643	[2.052, 4.571]	5.152	<0.001	[***]

*Note*: Ordinary least‐squares regression evaluating independent associations with conventional PTS. Positive coefficients indicate a greater, steeper PTS. Model 1 included the full cohort (*n* = 1406). Model 2 additionally included BMI in the complete‐case cohort (*n* = 1022); no observations other than those lacking BMI were omitted.

Positive *B* indicates greater (steeper) conventional PTS. Yellow = *p* < 0.05.

Abbreviations: ACL, anterior cruciate ligament; *B*, unstandardized coefficient in degrees; BMI, body mass index; CI, confidence interval; MPFL, medial patellofemoral ligament; n.s., not significant; OLS, ordinary least squares; PTS, posterior tibial slope; SE, standard error; *β*, standardized coefficient.

* *p* < 0.05

** *p* < 0.01

*** *p* < 0.001.

After adjustment for age and sex, primary ACL rupture was independently associated with a 2.52° greater conventional PTS than with ACL‐intact controls (standardized *β* = 0.358; *p* < 0.001), whereas ACL re‐rupture was associated with a 3.63° greater PTS (standardized *β* = 0.202; *p* < 0.001).

Age demonstrated a statistically significant but clinically negligible inverse association with conventional PTS (−0.019° per year; 95% CI, −0.036° to −0.003°; *p* = 0.024), corresponding to less than 1° across four decades. Pearson correlation also showed a weak inverse association (*r* = −0.141; *p* < 0.001). Sex was not independently associated with PTS (Table 4).

**Table 4:** Correlations between conventional PTS and demographic variables.

Variable	*N*	Pearson *r* with PTS	*p* (Pearson)	Spearman *ρ* with PTS	*p* (Spearman)	Sig.
Age (years)	1406	−0.1414	<0.001	−0.1390	<0.001	***
BMI (kg/m^2^)	1022	−0.0503	0.108	−0.0565	0.071	n.s.

*Note*: Correlation coefficients for conventional PTS. Positive coefficients indicate that greater covariate values are associated with a greater, steeper PTS; negative coefficients indicate an inverse association.

Bold/yellow = significant correlation. Full‐cohort analysis: *n* = 1406. BMI complete‐case analysis: *n* = 1022 (384 values unavailable).

Pearson *r* and Spearman *ρ* correlations with conventional PTS. Two‐tailed *p* values. Negative coefficients indicate that greater covariate values are associated with a lower conventional PTS.

Abbreviations: BMI, body mass index; n.s., not significant; PTS, posterior tibial slope.

When BMI was added to the complete‐case model (*n* = 1022), BMI was not independently associated with PTS, whereas the associations between ACL status and conventional PTS remained essentially unchanged.

Subgroup analyses demonstrated no clinically meaningful differences in PTS according to age category (<18 vs. ≥18 years), sex or injury laterality (Table 5).

**Table 5:** Subgroup analyses of conventional PTS according to demographic and injury‐related characteristics.

Subgroup	n_1_	Mean_1_ PTS (°)	SD_1_ (°)	n_2_	Mean_2_ PTS (°)	SD_2_ (°)	Δ PTS (°)	95% CI (°)	*t*	df	*p* (*t*)	Cohen's *d*	*U*	*p* (MW)	Sig.
A. Adolescents (<18 years) versus adults (≥18 years) [Group 1: Adolescents | Group 2: Adults]															
ACL‐intact controls [adolescents vs. adults]	162	7.78	4.24	695	7.19	2.72	0.593	[−0.09, 1.28]	1.702	192.9	0.089	0.167	50,587	0.044	n.s.
Primary ACL rupture [adolescents vs. adults]	189	9.81	3.15	308	9.98	3.05	−0.173	[−0.74, 0.39]	−0.601	387.9	0.548	−0.056	28,375	0.638	n.s.
ACL re‐rupture group excluded from the age‐category comparison (0 adolescents). Δ PTS = adolescents − adults.															
Welch *t* test and Mann–Whitney *U* test. Significance labels refer to the Welch *t* test results.															
B. Male versus female patients [Group 1: Male | Group 2: Female]															
ACL‐intact controls [male vs. female]	356	7.26	3.00	501	7.33	3.12	−0.067	[−0.48, 0.35]	−0.317	781.2	0.751	−0.022	87,070	0.555	n.s.
Primary ACL rupture [male vs. female]	249	9.88	3.29	249	9.97	2.88	−0.092	[−0.64, 0.45]	−0.332	487.5	0.740	−0.030	30,778	0.890	n.s.
ACL re‐rupture [male vs. female]	29	10.23	3.61	22	11.71	3.19	−1.475	[−3.35, 0.40]	−1.545	47.8	0.122	−0.433	242	0.143	n.s.
Δ PTS = male − female.															
C. Unilateral versus bilateral ACL injury [Group 1: Unilateral | Group 2: Bilateral]															
Primary ACL rupture [unilateral vs. bilateral]	395	9.97	3.02	15	9.31	4.14	0.660	[−1.46, 2.78]	0.611	14.6	0.541	0.182	2666	0.510	n.s.

*Note*: Conventional PTS, measured on lateral radiographs using the proximal anatomical tibial axis defined by two circles, was compared within study groups according to age category (<18 vs. ≥ 18 years), sex and ACL injury laterality (unilateral vs. bilateral). Larger PTS values indicate a steeper posterior slope. Values are reported as mean ± standard deviation. Mean differences were calculated as Group 1 minus Group 2 and are presented with 95% CIs, Welch *t* test statistics, Cohen's *d* effect sizes and Mann–Whitney *U* test results. All PTS values use the conventional definition; larger values indicate a steeper posterior slope. Bilateral ACL injury: *n* = 15. Primary ACL rupture group only.

Abbreviations: ACL, anterior cruciate ligament; BMI, body mass index; CI, confidence interval; n.s., not significant; PTS, posterior tibial slope; SD, standard deviation; *β*, standardized regression coefficient.

The final multivariable model explained 16.0% of the variance in PTS (*R* ^2 ^= 0.160; adjusted *R* ^2 ^= 0.158).

### ROC analysis

**Figure 3:**
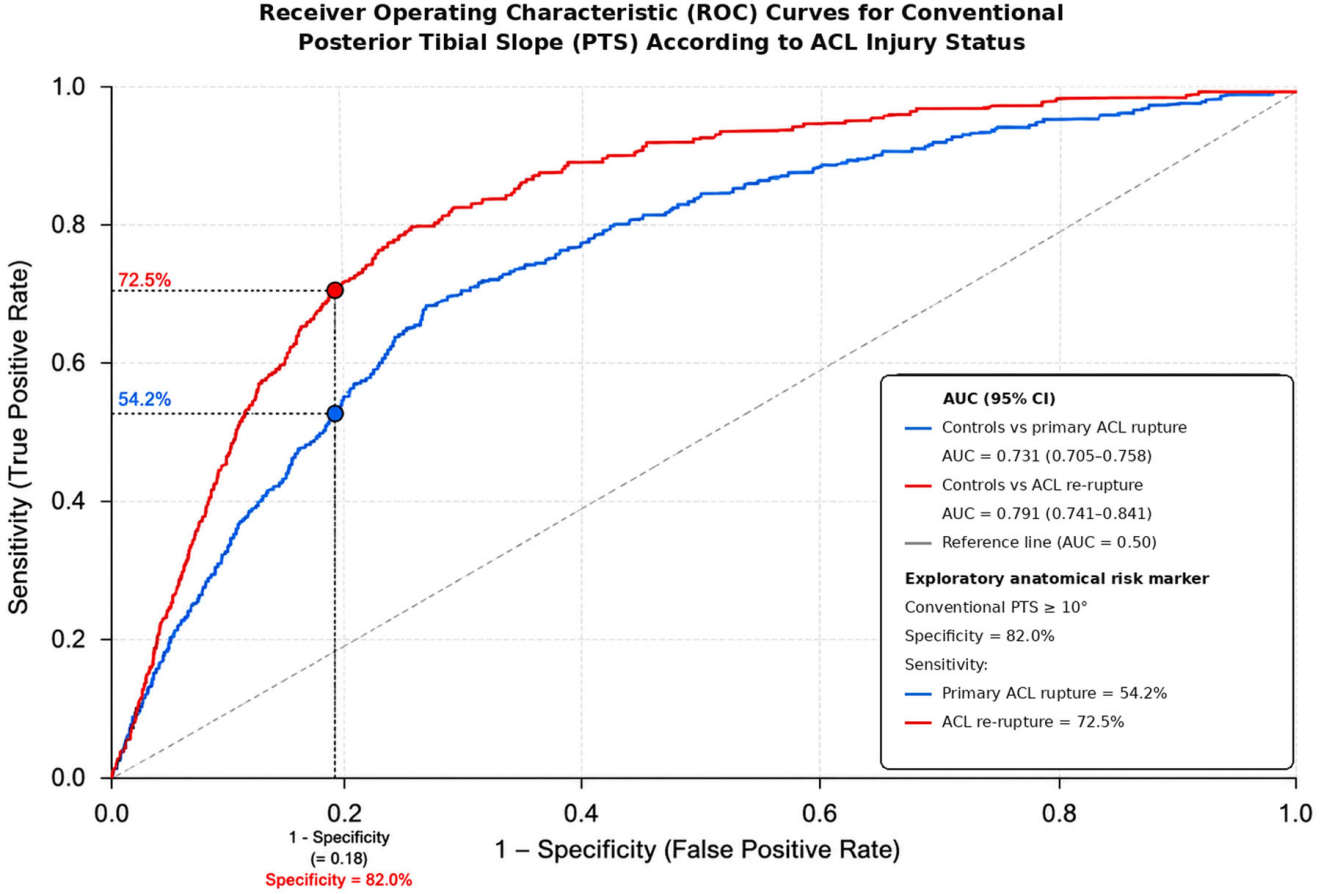
Receiver operating characteristic curves for conventional PTS according to ACL injury status. Receiver operating characteristic curves showing the ability of conventional PTS to distinguish ACL‐intact controls from patients with primary ACL rupture and ACL re‐rupture. The AUC was 0.731 (95% CI, 0.705–0.758) for primary ACL rupture and 0.791 (95% CI, 0.741–0.841) for ACL re‐rupture. A conventional PTS value of 10°, identified exploratorily using the Youden index, had a specificity of 82.0%, a sensitivity of 54.2% for primary ACL rupture and a sensitivity of 72.5% for ACL re‐rupture. Given the limited sensitivity, 10° should be interpreted only as a potentially specific anatomical risk marker and not as a diagnostic cutoff or screening threshold. ACL, anterior cruciate ligament; AUC, area under the curve; CI, confidence interval; PTS, posterior tibial slope.

ROC analysis demonstrated moderate discriminative performance of PTS for ACL injury (Figure 3).

The area under the curve (AUC) was 0.731 (95% CI, 0.705–0.758) for primary ACL rupture and 0.791 (95% CI, 0.741–0.841) for ACL re‐rupture.

The Youden‐derived value was 10° of PTS. Specificity was 82.0%, whereas sensitivity was 54.2% for primary ACL rupture and 72.5% for ACL re‐rupture.

The 10° value therefore represents a potentially specific anatomical risk marker and not a diagnostic or screening threshold.

## DISCUSSION

The principal finding was that ACL injury status was independently associated with PTS, whereas age, sex and BMI had no clinically meaningful independent influence. The association persisted after adjustment for demographic variables in a large cohort including ACL‐intact controls, patients with primary ACL rupture and patients with ACL re‐rupture.

Patients with primary ACL rupture and ACL re‐rupture demonstrated greater PTS than ACL‐intact controls. This finding is consistent with previous clinical and systematic‐review evidence showing that increased PTS is associated with both ACL rupture and graft failure after ACL reconstruction [13, 21]. Beel et al. reported a high prevalence of increased PTS in patients undergoing revision ACL reconstruction, using a threshold of 12° to define increased PTS [1]. Dean et al., in a systematic review and meta‐analysis, also reported greater PTS in patients with failed ACL grafts compared with patients with primary ACL tears or intact ACLs [3]. These findings support the concept that PTS is part of an anatomical risk profile for ACL injury and recurrent instability.

PTS should not be interpreted as a standalone screening test. The ROC analysis identified a Youden‐derived value of 10° with good specificity but limited sensitivity for primary ACL rupture. This value may represent a potentially specific anatomical risk marker, but it is not a screening threshold [5, 14, 15]. ACL injury is multifactorial, and osseous morphology interacts with neuromuscular control, sport exposure, activity level, ligamentous laxity, meniscal morphology, coronal alignment and injury mechanism.

No clinically meaningful independent association was observed between demographic variables and PTS. Age showed a very small inverse effect, corresponding to less than 1° across four decades, whereas sex and BMI were not independently associated with PTS. Thus, standardized radiographic PTS may be interpreted without routine demographic adjustment. Prior studies have reported variation in PTS according to sex, age, race and measurement modality [19, 21]. In this cohort, ACL status had a stronger association with PTS than these demographic characteristics.

Measurement technique is central to PTS interpretation. Radiographic and MRI‐based methods use different reference axes and can produce systematically different values and thresholds [6, 16]. A single standardized radiographic technique was therefore used throughout the cohort. All values, ranges, group differences, regression coefficients, correlations, tables, legends and figure labels are reported using conventional PTS, in which larger values indicate a steeper posterior slope.

Radiographic quality is critical for reliable PTS assessment. Only true lateral radiographs fulfilling predefined quality criteria were included, and images with malrotation or inadequate condylar superimposition were excluded. Intraobserver and interobserver reliability were good to excellent and moderate to good, respectively. However, reliability is not equivalent to accuracy; accuracy against an external reference standard and absolute measurement error were not assessed, and residual error related to radiographic positioning cannot be excluded.

Several limitations apply. The retrospective single‐centre design may limit generalizability. The cross‐sectional design precludes causal inference, and whether the observed PTS differences preceded injury cannot be determined. PTS was measured on lateral radiographs using the proximal anatomical tibial axis defined by two circles; compartment‐specific medial and lateral slopes were not analysed separately [5]. The MPFL group was a surgical rather than population‐based comparator, although ACL integrity was confirmed on MRI. The ACL re‐rupture group was small (*n* = 51), limiting precision. Baseline age differed between groups, and residual confounding by activity or injury mechanism cannot be excluded. BMI was available for 1022 of 1406 patients (72.7%); BMI‐adjusted analyses were restricted to this complete‐case subset. Activity level was unavailable. Standard lateral radiographs remain susceptible to positioning and rotation. Finally, accuracy against an external reference standard and absolute measurement error were not assessed.

## CONCLUSION

PTS was independently associated with ACL injury and ACL re‐rupture, whereas age, sex and BMI had no clinically meaningful independent influence. PTS should be interpreted as one component of a multifactorial anatomical risk profile rather than as a standalone screening test.

## AUTHOR CONTRIBUTIONS

Julien Druel contributed to study conception and design, data collection, radiographic measurements, statistical analysis and manuscript drafting. Antoine Piercecchi and Lyes Chaal contributed to data collection, radiographic measurements and manuscript revision. Christophe Jacquet and Romir Patel contributed to data interpretation, methodological supervision and critical revision of the manuscript. Matthieu Ollivier supervised the study, contributed to study design, data interpretation and critical revision of the manuscript.

## FUNDING INFORMATION

The authors have no funding to report.

## CONFLICT OF INTEREST STATEMENT

The authors declare no conflicts of interest.

## ETHICS STATEMENT

This study was approved by the local Institutional Review Board (Comité d'éthique de l'Institut du Mouvement et de l'Appareil Locomoteur, Aix‐Marseille Université) under approval number IRB‐2023‐ACL‐07.

## Data Availability

The data that support the findings of this study are available from the corresponding author upon reasonable request. Due to institutional and ethical restrictions, the data are not publicly available.
